# Coto Bark

**Published:** 1879-10

**Authors:** 


### COTO BARK.

Prof. Fronmüller and Prof. Balz have been experimenting therapeutically with coto bark, a plant whose botanical position is not yet settled by naturalists. The powdered bark and a tincture made of one part bark to nine of alcohol were employed, the quantity of the latter used varying from 0.50 grm. to 25.00 grms. daily. Out of eighty-five cases of colliquative diarrhœa, fifty were cured, twenty-six were benefited, and nine showed no results after the administration of the drug. In many cases the diarrhœa returned within a few days, but a repetition of the treatment effected a final cure. As a rule, the success was in direct proportion to the dose, the medicine failing when small doses were used. The profuse sweating of phthisis was also controlled by it in a remarkable degree, and of ninety-one observations recorded by Fronmüller, it disappeared entirely in thirty-four, was diminished in twenty-six, and in eight was not effected. An alkaloid, termed cotoine, is obtained from the bark by means of ether, which may be used instead of the substance itself, 0.15 grm. being equal to 5.00 grms. of the tincture. Another crystalline body, paracotoine, is also obtained, and was used by Prof. Balz in cholera; but owing to the small quantity of the drug in his possession, he was unable to form a satisfactory opinion of its action; the results, however, were sufficiently favorable to recommend its further trial.—*Bulletin General de Therapeutique, April, 1879.*

